# Dual resistance to *Flavobacterium psychrophilum* and *Myxobolus cerebralis* in rainbow trout (*Oncorhynchus mykiss*, Walbaum)

**DOI:** 10.1111/jfd.13605

**Published:** 2022-03-08

**Authors:** Brian W. Avila, Dana L. Winkelman, Eric R. Fetherman

**Affiliations:** ^1^ Colorado Cooperative Fish and Wildlife Research Unit Colorado State University Fort Collins Colorado USA; ^2^ U.S. Geological Survey Colorado Cooperative Fish and Wildlife Research Unit Department of Fish, Wildlife and Conservation Biology Colorado State University Fort Collins Colorado USA; ^3^ Colorado Parks and Wildlife Fort Collins Colorado USA

**Keywords:** bacterial coldwater disease, *Flavobacterium psychrophilum*, *Myxobolus cerebralis*, rainbow trout, whirling disease

## Abstract

Aquatic pathogens are a major concern for fish hatchery production, fisheries management, and conservation, and disease control needs to be addressed. Two important salmonid pathogens are *Myxobolus cerebralis* and *Flavobacterium psychrophilum* that cause whirling disease and bacterial coldwater disease (BCWD), respectively. Innate disease resistance is a potential option for reducing disease‐related mortality in hatchery‐reared rainbow trout (*Oncorhynchus mykiss*, Walbaum). Two experiments were conducted to assess pathogen resistance of first‐generation (F1) rainbow trout created by crossing *M. cerebralis*‐ and *F. psychrophilum*‐resistant strains. In the first experiment, we exposed two rainbow trout strains and one F1 cross to six treatments: control (no exposure), mock injection, *F. psychrophilum* only, *M. cerebralis* only, *F. psychrophilum* then *M. cerebralis*, and *M. cerebralis* then *F. psychrophilum*. Results indicated that the F1 cross was not resistant to either pathogen. In the second experiment, we exposed five rainbow trout strains and four rainbow trout crosses to *F. psychrophilum*. The second experiment indicated that at least one rainbow trout cross was *F*. *psychrophilum*‐resistant. Achieving dual resistance may be possible using selective breeding but only some multigenerational strains are suitable candidates for further evaluation.

## 1 INTRODUCTION

*Flavobacterium psychrophilum*, the causative agent of bacterial coldwater disease (BCWD), is found in cultured and wild fishes worldwide and causes significant infection in captive salmonid populations (LaFrentz & Cain, 2004; Starliper, 2011). Mortality associated with infections can be as high as 90% (Barnes & Brown, 2011; Nilsen et al., 2011) depending on water temperature and developmental stage of the host (Decostere et al., 2001; Wood, 1974). Outbreaks causing high mortality can result in massive economic losses to producers of salmon and rainbow trout (*Oncorhynchus mykiss*, Walbaum) (Antaya, 2008). As a result, BCWD is considered one of the most important hatchery diseases in the world (Michel et al., 1999). Infections typically affect age‐0 salmonids (Cipriano & Holt, 2005; Nicolas et al., 2008) but can also affect larger and older fish (LaFrentz & Cain, 2004). Infected fish show a broad range of clinical disease signs such as discoloration of the adipose fin, lesions, spiral swimming behaviour, “blacktail”, spinal deformities, and pale or necrotic gills (Borg, 1948; Davis, 1946; Kent et al., 1989; Martinez et al., 2004; Ostland et al., 1997).

Antibiotics are the most used treatment for an *F. psychrophilum* infection. Oxytetracycline (OTC) has been used worldwide (Branson, 1998; Groff & LaPatra, 2001; LaFrentz & Cain, 2004; Lumsden et al., 2006; Post, 1987), and amoxicillin and oxolinic acid have been used throughout Europe (Branson, 1998; Bruun et al., 2000). Several studies suggest that antimicrobial resistance is occurring in treated populations (Bruun et al., 2000; Schrag & Wiener, 1995). Starting in 1986, oxolinic acid was used to treat *F. psychrophilum* in Denmark hatcheries, but by 2000, the bacteria were 100% resistant to this treatment. Between 1994 and 1998, 60%–75% of *F. psychrophilum* in Danish hatcheries showed resistance to both OTC and amoxicillin (Bruun et al., 2000). Another potential treatment option for BCWD is vaccination, and though development of a vaccine has been attempted (Sudheesh & Cain, 2016), none are currently commercially available.

Due to concerns about antibiotic resistance and the lack of a vaccine, other strategies to prevent *F. psychrophilum* infections warrant investigation. Hadidi et al. (2008) suggested using a genetically resistant brood fish to manage BCWD outbreaks. In 2005, the US Department of Agriculture‐Agricultural Research Service's (USDA‐ARS) National Center for Cool and Cold Water Aquaculture (NCCWA) developed a program to create a rainbow trout strain that was genetically resistant to *F. psychrophilum* (Hadidi et al., 2008; Leeds et al., 2010). The strains used to create the *F. psychrophilum*‐resistant population were chosen based on known genetic and domestication history including the Ennis National Fish Hatchery Shasta strain; College of Southern Idaho, House Creek strain; Kamloops/Puget Sound Steelhead cross; and University of Washington, Donaldson strain (Silverstein et al., 2009). The resulting *F. psychrophilum*‐resistant rainbow trout, the ARS‐Fp‐R strain, showed reduced mortality when exposed to *F. psychrophilum* (Leeds et al., 2010; Wiens et al., 2013). A third‐generation lot of ARS‐Fp‐R was sent to Utah Division of Wildlife Resources (UDWR), and then in 2016, Colorado Parks and Wildlife (CPW) imported the *F. psychrophilum*‐resistant rainbow trout from UDWR to be used in the CPW hatchery system to manage mortality due to *F. psychrophilum* infections. Within the CPW hatchery system, these imported fish are known as *psychrophilum*‐resistant rainbow (PRR). As of 2020, the USDA‐ARS NCCWA had produced its fifth generation of *F. psychrophilum*‐resistant rainbow trout (G. Weins, USDA personal communication, June 18, 2019). A similar approach has been used to produce whirling disease *Myxobolus cerebralis* resistant rainbow trout and reestablish rainbow trout fisheries in the presence of the parasite (Fetherman et al., 2011, 2012, 2014; Schisler et al., 2006).

Using *F. psychrophilum*‐resistant fish in hatcheries system may provide a valuable tool to reduce mortality due to outbreaks of BCWD, and *F*. *psychrophilum*‐resistant rainbow trout, or PRR, were brought into the Colorado's hatchery system in 2016 for that purpose. However, it is unknown whether the PRR are also resistant to *M. cerebralis*. *M. cerebralis* was introduced to Colorado in the late 1980s, and later found in free‐ranging salmonid populations in 11 of the state's 15 major river drainages (Barney et al., 1988; Nehring & Thompson, 2003), resulting in the collapse of wild rainbow trout populations throughout Colorado (Nehring & Thompson, 2001). The state of Colorado has been using *M. cerebralis*‐resistant rainbow trout to re‐establish populations in the presence of the parasite (Avila et al., 2018; Fetherman et al., 2014), and reproduction and recruitment are occurring (Fetherman et al., 2014). Stocking *F. psychrophilum*‐resistant fish with no resistance to *M. cerebralis* could result in failure due to mortality associated with *M. cerebralis* exposure, as well as increased infection severity and loss of progress gained from *M. cerebralis*‐resistant rainbow trout stocking efforts. For *F. psychrophilum*‐resistant fish to be a viable management tool, it is imperative to determine if the PRR exhibit any resistance to *M. cerebralis*, understand if resistance to both *F. psychrophilum* and *M. cerebralis* is compatible and achievable, and, given the possibility for exposure to either or both pathogens in a hatchery or wild environment, understand how they might interact when dual exposure occurs.

Our overall goal was to determine if crossing strains of rainbow trout resistant to each pathogen would result in progeny that were genetically resistant to both pathogens, and this was evaluated using two experiments. The goal of the first experiment was to determine if it was possible to develop dual resistance by crossing a rainbow trout resistant to *M. cerebralis* with the PRR. We also wanted to understand the possible effects of coinfection when fish were exposed to both pathogens and if the order of exposure was important. Fish could be infected with *F. psychrophilum* in the hatchery and then be stocked into the wild and exposed to *M. cerebralis*. Conversely, in an *M. cerebralis*‐positive hatchery, fish may be exposed to *M. cerebralis* first followed shortly after by *F. psychrophilum* exposure. The goal of our second experiment was to determine if it was possible to develop a first‐generation (F1) rainbow trout cross that was resistant to *F. psychrophilum* when crossing pure parental strains resistant to either *F. psychrophilum* or *M. cerebralis*.

## 2 METHODS

### 2.1 Rainbow trout strains and crosses

Three strains of *M. cerebralis*‐resistant rainbow trout were used for both experiments, German rainbow trout, Harrison Lake rainbow trout, and the German Rainbow × Harrison Lake rainbow trout (Table 1). The pure German Rainbow (GR) is a domesticated hatchery rainbow trout which was exposed to *M. cerebralis* over many generations in Germany and is more resistant to *M. cerebralis* than many other rainbow trout strains found in North America (Hedrick et al., 2003). The Harrison Lake rainbow trout (HL; origin: Harrison Lake, Montana; Wagner et al., 2006) is one of the wild rainbow trout strains that were crossed with the GR to create a fish capable of surviving and reproducing in the wild (Fetherman et al., 2015; Schisler & Fetherman, 2009). The GR × HL used within Experiment 1 are 87.5% GR and 12.5% HL and has been propagated as a hatchery brood stock since 2006 (Schisler et al., 2011). Despite showing *M. cerebralis* resistance, the GR × HL shows some of the highest mortality in the CPW hatchery system due to *F. psychrophilum* infections. In both experiments, we used the PRR from Colorado and the fifth‐generation ARS‐Fp‐R from the USDA‐ARS NCCWA to investigate *F. psychrophilum* resistance (Table 1).

**TABLE 1:** Rainbow trout strains used for each experiment and their known pathogen resistance

Rainbow Trout Strains/Crosses	Abbreviation	Experiment	Resistance	Fish type
German Rainbow × Harrison Lake	GR × HL	1	*M. cerebralis*	Strain
(German Rainbow × Harrison Lake) × *psychrophilum*‐resistant rainbow	GHP	1	Unknown	F1‐generation cross
*psychrophilum*‐resistant rainbow	PRR	1, 2	*F. psychrophilum*	Strain
Harrison Lake	HL	2	*M. cerebralis*	Strain
Germain Rainbow	GR	2	*M. cerebralis*	Strain
S‐Line	ARS‐Fp‐S	2	Unknown	Strain
Agricultural Research Service ‐ *F. psychrophilum* ‐ Resistant	ARS‐Fp‐R	2	*F. psychrophilum*	Strain
Harrison Lake × *psychrophilum*‐resistant rainbow	HL × PRR	2	Unknown	F1‐generation cross
Harrison Lake × Agricultural Research Service ‐ *F. psychrophilum* ‐ Resistant	HL × ARS‐Fp‐R	2	Unknown	F1‐generation cross
German Rainbow × *psychrophilum*‐resistant rainbow	GR × PRR	2	Unknown	F1‐generation cross
German Rainbow × Agricultural Research Service ‐ F. psychrophilum ‐ Resistant	GR × ARS‐Fp‐R	2	Unknown	F1‐generation cross

### 2.2 Experiment 1 – dual exposure to *Flavobacterium psychrophilum* and *Myxobolus cerebralis*

**FIGURE 1:**
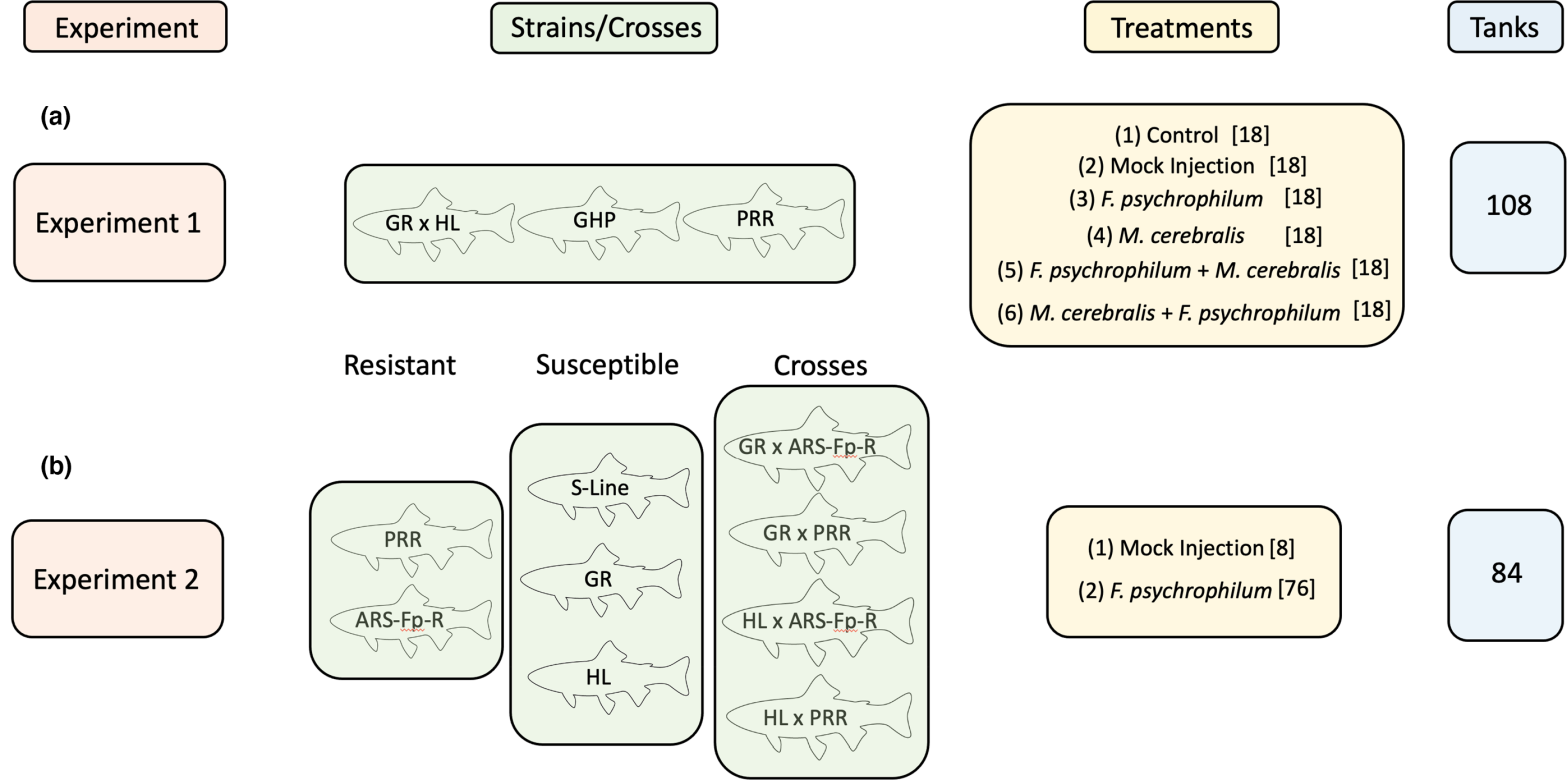
Experimental design for Experiment 1 (a) and Experiment 2 (b). Three strains or crosses were used in Experiment 1. Each strain was exposed to six treatments, with the number of tanks for each treatment denoted by [ ] for a total of 108 tanks. Nine strains or crosses were used in Experiment 2 and evaluated only for resistance to *F*. *psychrophilum* using two treatments and a total of 84 tanks

Two strains and one cross of rainbow trout were used for this dual exposure experiment (Table 1; Figure 1), the PRR, which is resistant to *F. psychrophilum*, the GR × HL, which is resistant to *M. cerebralis,* and the cross of the GR × HL and PRR (GHP), which was created and evaluated for maintaining resistance to both pathogens. These strains were spawned at the CPW Crystal River Hatchery (Carbondale, Colorado) in January 2019 and then transported as eyed eggs to the CPW Bellvue Fish Research Hatchery (Bellvue, Colorado) for hatching. Fish were moved from the CPW Bellvue Fish Research Hatchery, at eight weeks post‐hatch to a laboratory located on the Colorado State University (CSU) main campus. Fish were moved two days prior to the beginning of the experiment.

The PRR, GR × HL, and GHP were exposed to six treatments: (1) no pathogen exposure (control), (2) mock injection, (3) *F. psychrophilum*‐only exposure, (4) *M. cerebralis*‐only exposure, (5) *F. psychrophilum* exposure followed by *M. cerebralis* exposure four days later, and (6) *M. cerebralis* exposure followed by *F. psychrophilum* exposure four days later. Each strain and treatment combination had six replicates, resulting in 108 total twenty‐gallon (76‐L) tanks. Thirty‐five individual rainbow trout of the assigned strain/cross were contained in each tank, resulting in 3780 fish in the experiment. Water (13.4ºC ± 2.1 SD) was sourced from the city and dechlorinated by running through large, activated charcoal filters. Tanks were set up for flow‐through water exchange at a flow of 15 gallons per hour.

To limit potential cross‐contamination from pathogen‐exposed tanks, control tanks were located on the top shelf of the three‐tier shelving system. Strains were randomly assigned to tanks and either a control or mock injection treatment within the top shelf of the system. Pathogen exposure treatments and strains were then randomly assigned to the remaining tanks on the top shelf (note that control/mock injection tanks were never located next to pathogen exposure tanks on the same shelving unit) and the tanks on the other two tiers of the shelving system.

*Flavobacterium psychrophilum* culture and preparation are described in Avila (2021). An initial batch weight of the fish in each tank was taken and used to calculate the amount of feed per day (g) and the dose of *F. psychrophilum* given the average weight per fish (PRR: 0.46 g ± 0.03 SD; GR × HL: 0.41 g ± 0.05; GHP: 0.48 g ± 0.06). For *F. psychrophilum* exposure, rainbow trout were first sedated using MS‐222 (90 mg/ml of water) and then injected subcutaneously at the dorsal midline posterior to the dorsal fin with 8.8 × 10^6^ colony forming units per millilitre (CFU/ml; 25 μl) of virulent *F. psychrophilum* (CSF259‐93 obtained from K. Cain, Moscow, Idaho). For the mock injection, rainbow trout were similarly subcutaneously injected with 25 μl of tryptone yeast extracts and salt (TYES) to verify that exposure to the bacteria and not physical injury from injection caused mortality. No injections occurred for fish in the control, *M. cerebralis* only, or, initially, the *M. cerebralis* exposure followed by *F. psychrophilum* treatments.

*Myxobolus cerebralis* triactinomyxons (TAMs), the waterborne infectious stage of the parasite, were produced by *Tubifex tubifex* worm cultures maintained at the CPW Parvin Lake Research Station (Red Feather Lakes, Colorado). The concentration of viable TAMs was estimated by mixing 1000 μl of filtrate containing TAMs and 60 μl of crystal violet; 84.6 μl of this mixture was then placed on a slide and the number of TAMs per slide was counted. Ten TAM counts were conducted out of the filtrate to get an average number of TAMs per mL, and fish (732.2 ± 34.6 degree‐days [°C*days] post‐hatch) were exposed to 2,000 TAMs per individual for a total of 70,000 TAMs per tank (following Fetherman et al., 2011). Fish in the control treatment, mock injection, *F. psychrophilum* only, and, initially, *F. psychrophilum* exposure followed by *M. cerebralis* exposure were not exposed to TAMs.

On the first day of the experiment (day zero), mock injections were conducted first to prevent accidental exposures to *F. psychrophilum* using the same injectors, followed by *F. psychrophilum* treatments (*F. psychrophilum* only, *F. psychrophilum* and then *M. cerebralis*) and *M. cerebralis* treatments (*M. cerebralis* only, *M. cerebralis* and then *F. psychrophilum*). The same exposure methods described above were used for the dual exposures, but some (*n* = 18 tanks each) were exposed to *F. psychrophilum* and then *M. cerebralis* four days later or exposed to *M. cerebralis* and then *F. psychrophilum* four days later.

Experiment 1 had two objectives. The first was to conduct an *F. psychrophilum* exposure experiment to observe mortality. *Flavobacterium psychrophilum* exposures were conducted on day zero and day four and fish were held for 28 days so that mortality could stabilize (showing no more mortality) because fish can survive and recover from *F. psychrophilum* infections. The second objective started after the completion of the *F. psychrophilum* portion of the experiment. All remaining fish were reared until they reached 2347.8 ± 73.3 SD degree‐days, which was necessary to ensure full development of myxospores in the treatments with *M. cerebralis*.

Tanks were cleaned every two weeks on a rotating schedule. Throughout the rearing process all tanks were monitored twice daily and moribund and dead fish in each tank were measured, weighed, signs of disease were documented, and the fish were then removed. Fish were fed twice a day at the standardized feeding rate (per cent body weight per day [% BW/d]) based on the manufacturer's (BioOregon) suggested specifications of fish size and rearing temperature. Feed amount was adjusted daily based on the number of fish within each tank. Batch weights used to adjust feeding rates were taken from each tank by placing all fish from the tank into a tared water bucket on a scale, obtaining individual weights by dividing the total weight by the known number of fish, and calculating the grams per fish. Batch weighing was conducted every two weeks starting 28 days post‐exposure to prevent affecting mortality results in the classic *F. psychrophilum* exposure experiment by handling fish.

After reaching the required degree days for myxospore development, all surviving fish were euthanized, weighed, measured, and inspected for clinical signs of whirling disease and/or BCWD. Euthanized fish had their heads removed from the body just behind the operculum and pectoral fins and placed in individually labelled bags and frozen (Fetherman et al., 2012). Myxospores were enumerated (O’Grodnick, 1975) using pepsin–trypsin digest (PTD; Markiw & Wolf, 1974a, 1974b). The processing of fish was initiated at the CPW Aquatic Animal Health Laboratory (AAHL; Brush, Colorado) and conducted by the AAHL staff. A subset of samples were processed entirely by the AAHL, including pepsin‐trypsin digestion and myxospore counting. The remaining fish were digested using pepsin at the AAHL and then transferred to the Colorado Cooperative Fish and Wildlife Research Unit laboratory to finish trypsin digestion and myxospore counting. The same methods were used in both laboratories to ensure consistency in the results.

#### 2.2.1 Statistical analysis

The statistical analysis focused on five endpoints: (1) 28‐day post‐exposure mortality, (2) end of experiment mortality, (3) differences in growth, (4) disease signs, and (5) myxospore counts for *M. cerebralis*‐exposed tanks.

#### 2.2.2 Mortality

Cumulative per cent mortality (CPM), the number of dead fish divided by the total number of fish at the start, was calculated for each tank for the first 28 days post‐exposure and at the end of the experiment. A chi‐squared test was used to determine if there were differences in mortality (a proportion) among the strains, treatments, and strain by treatment interaction (strain*treatment). If there was evidence of a difference in mortality, pairwise comparisons with a Tukey adjustment were used to compare among strains and treatments.

#### 2.2.3 Growth

The difference in weight at 28 days post‐exposure was analysed using ANOVA with strain, treatment, and strain*treatment as the factors to explain differences in growth due to exposure to *F. psychrophilum*. The difference in weight at the end of the experiment was analysed using an ANCOVA with strain, treatment, and interaction between strain and treatment as the factors, and the number of fish within a tank at the end of the experiment as a covariate as tank density was thought to explain differences in growth. The difference in weight at the end of the experiment may also show if growth was affected by surviving *F. psychrophilum* infection because traditional *F. psychrophilum* exposure experiments are only conducted for 28 days. If there was evidence of a difference in growth in either model, then a pairwise comparison with a Tukey adjustment was implemented. Type III sums of squares were used to account for the unbalanced number of tanks and statistical significance was inferred at the α = 0.05 level.

#### 2.2.4 Myxospore counts

To account for myxospore counts of zero in the control, mock injection, and *F. psychrophilum*‐only exposure, a two‐part modelling approach was taken. First, we used a logistic regression to quantify the difference in myxospore counts between the tanks not exposed to *M. cerebralis* to those that were exposed to *M. cerebralis*. The response is specified by a binary variable (0 if not exposed to *M. cerebralis* or 1 if a fish was exposed to *M. cerebralis*) with the predictor variables of strain, treatment, and the strain by treatment interaction. Chi‐squared values were then used to determine if there were statistical differences in the number of myxospores between the predictor variables. Second, we used a negative‐binomial regression, which helped account for overdispersion of the data (Boulton & Williford, 2018; Duan et al., 1983), to compare the average difference in myxospore count among strains, treatments, and their interaction in only fish that were exposed to *M. cerebralis*. If there was evidence of differences in myxospore counts, then a pairwise comparison with a Tukey adjustment was used to compare among strains and treatments.

#### 2.2.5 Clinical signs of whirling disease

Clinical whirling disease signs included cranial deformities, spinal deformities, opercular deformities, exophthalmia, lower jaw deformities, and blacktail. We observed bubbles on the fins and near the gills on some individuals, indicating the potential for gas bubble disease at the end of the experiment. No additional effects of mortality were observed due to gas bubble disease; however, exophthalmia was removed from the analysis because it might have been due to gas bubble disease and not exposure to *M. cererbralis*. A logistic regression was used to fit the data with the proportion of individuals in a tank as the response and strain, treatment, and the interaction between strain and treatment as the factors that predict clinical signs of disease. If there was evidence of a difference in clinical signs of disease, then a pairwise comparison with a Tukey adjustment was used to compare among strains and treatments.

### 2.3 Experiment 2

F1‐generation crosses were created by crossing pure German Rainbow (GR) and pure Harrison Lake rainbow trout (HL) with ARS‐Fp‐R obtained from the USDA‐ARS NCCWA or PRR obtained from CPW. All strains were created in collaboration with the USDA‐ARS NCCWA, the CPW Crystal River Hatchery, and the CPW Bellvue Fish Research Hatchery. The NCCWA provided ARS‐Fp‐R milt and the Crystal River Hatchery provided PRR milt, and milt from these sources were crossed with pure GR and pure HL eggs at the Bellvue Fish Research Hatchery in January 2020. All F1 crosses were made using *F. psychrophilum*‐resistant males and *M. cerebralis*‐resistant females. The spawning resulted in first‐generation HL × PRR, HL × ARS‐Fp‐R, GR × PRR, and GR × ARS‐Fp‐R crosses (Table 1; Figure 1). The Crystal River Hatchery produced pure PRR rainbow trout, whereas the NCCWA produced the ARS‐Fp‐R and an *F. psychrophilum‐*susceptible line rainbow trout (S‐Line), and both facilities shipped eyed eggs to the Bellvue Fish Research Hatchery where they were hatched.

Fish were moved from the Bellevue Fish Research Hatchery to a laboratory located on the CSU main campus. An initial sample weight of each tank was taken prior to moving fish to obtain average individual fish weights for each strain or cross (HL: 0.78 g ± 0.06 (*SD*); HL × PRR: 1 g ± 0; HL × ARS‐Fp‐R: 1 g ± 0; GR: 1 g ± 0; GR × PRR: 1.18 g ± 0.06; GR × ARS‐Fp‐R: 1.24 g ± 0.09; PRR: 1.46 g ± 0.10; S‐Line: 1.1 g ± 0.14; ARS‐Fp‐R: 1.1 g ± 0.12). Weights were used to calculate the total amount of feed per day (g) for each tank and *F. psychrophilum* dosage. Control fish were randomly assigned to tanks located on the top shelf of the three‐tier shelving system to limit potential bacterial contamination of control tanks, and *F. psychrophilum* treatment tanks were randomly assigned to the remaining tanks on the lower two tiers. Each twenty‐gallon (76‐L) tank held twenty‐five individual rainbow trout of the assigned strain/cross. Water (10°C ± 0.77 SD) was sourced from the city and dechlorinated by running through large, activated charcoal filters. Fish tanks were set up for flow‐through water exchange at a flow of 15 gallons per hour.

We compared five pure strains of rainbow trout, GR, HL, ARS‐Fp‐R, PRR and the S‐Line, and four F1‐generation crosses, GR × ARS‐Fp‐R, HL × ARS‐Fp‐R, GR × PRR and HL × PRR. All nine were injected subcutaneously posterior to the dorsal fin above the midline with virulent 8.8x10^6^ CFU/mL of *F. psychrophilum* (CSF259‐93; 25 μl), with ten replicates (tanks) each except for the GR × ARS‐Fp‐R (five tanks) which had high mortalities in the hatchery, and the S‐Line rainbow trout (two tanks). Mock injections were given to four strains (GR, HL, ARS‐Fp‐R and HL × ARS‐Fp‐R), with two replicates each, and injected similarly with TYES (25 μl). We did not include mock injections for every strain or equal numbers of replicates because water resources were limited in the laboratory. Fish were monitored twice a day after injections. Moribund and dead fish were removed from each tank and recorded for 28‐days after injection. At the end of the rearing period, all remaining fish were euthanized.

#### 2.3.1 Statistical analysis

Cumulative per cent mortality (CPM) was calculated for each tank at the end of the 28‐day experiment. A chi‐squared test was used to determine if there was a relationship between CPM and strain, exposure, and the interaction between strain and exposure (strain*exposure) and if there was a relationship between mortality and strain dependent on fish weight (strain*weight). If there was evidence of a difference in mortality, then Tukey adjusted pairwise comparisons were used to compare among strains and treatments. Finally, a logistic regression was used to estimate the probability of mortality based on factors that significantly affected CPM identified in the chi‐square analysis.

## 3 RESULTS

### 3.1 Experiment 1

#### 3.1.1 Mortality

For all three strains, control and TYES treatment CPMs were low (0‐‐10%) and did not differ at 28 days post‐exposure. The single exposures to *F. psychrophilum* only and *M. cerebralis* only resulted in CPMs ranging from 32%–90% and 0%–1% at 28 days post‐exposure, respectively. The CPMs for the dual exposures, *F. psychrophilum* followed by *M. cerebralis* exposure and *M. cerebralis* followed by *F. psychrophilum* exposure, ranged from 46%–94% and 58%–99%, respectively. The chi‐squared test indicated an interaction between strain and treatment (χ^2^ = 70.17, *p*‐value <.01). Mortality at 28 days was significantly higher in *F. psychrophilum* exposure treatments compared to controls, TYES, and *M. cerebralis* treatments. Mortality did not differ between the GR × HL and GHP when exposed to *F. psychrophilum*. However, mortality for the PRR was lower than either the GR × HL or GHP when exposed to *F. psychrophilum*.

**FIGURE 2:**
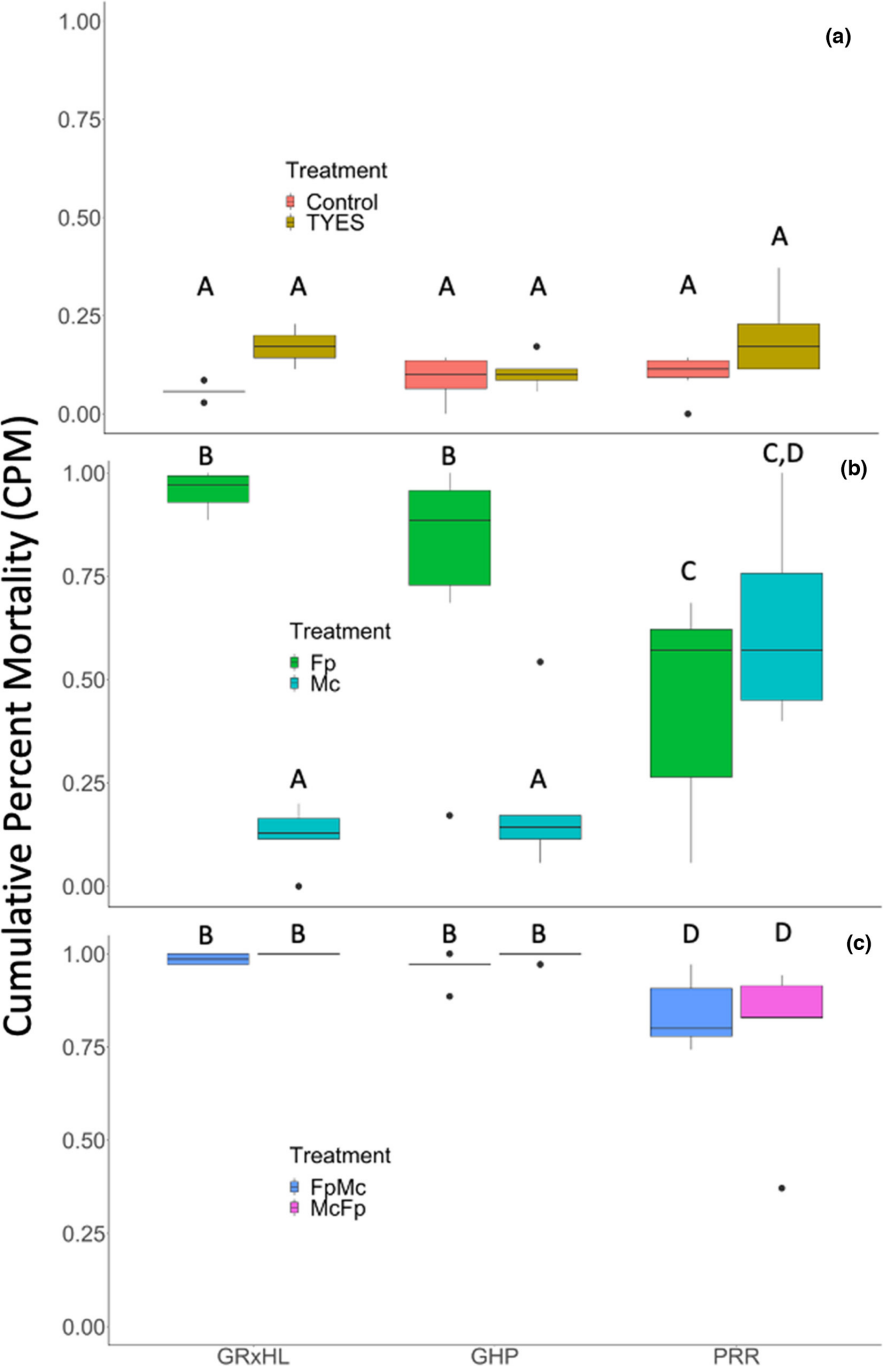
Cumulative per cent mortality (CPM) by strain and treatment at the end of Experiment 1. (a) Control and mock injection (TYES), (b) *Flavobacterium psychrophilum* only (Fp) and *Myxobolus cerebralis* only (Mc), and (c) *F. psychrophilum* followed by *M. cerebralis* (FpMc) and *M. cerebralis* followed by *F. psychrophilum* (McFp). Black lines within the boxes indicate the median of the distribution. Box and whisker plots with the same letter indicate no significant differences and box and whisker plots with different letters indicate statistically significant differences

Mean mortality at the end of the experiment ranged from 9% to 100% (Figure 2). The chi‐squared test indicated an interaction between strain and treatment (χ^2^ = 428.12, *p*‐value <.01). The control and TYES treatments showed low mortality and did not differ among strains (Figure 2a). *Flavobacterium psychrophilum‐*only exposure caused high mortality in GR × HL and GHP but was significantly lower in PRR, indicating that dual resistance was not achieved (Figure 2b). *Myxobolus cerebralis*‐only exposure caused high mortality in PRR but was significantly lower in GRR and GR × HL (Figure 2b), indicating that the PRR is susceptible to *M. cerebralis*. Mortality in GRR and GR × HL exposed to *M. cerebralis* was not distinguishable from the control or TYES treatments (Figure 2a,b). Dual exposures resulted in higher mortality compared to *F. psychrophilum*‐only and *M. cerebralis*‐only exposures for the PRR but not the GR × HL or GHP (Figure 2).

#### 3.1.2 Myxospore counts

**FIGURE 3:**
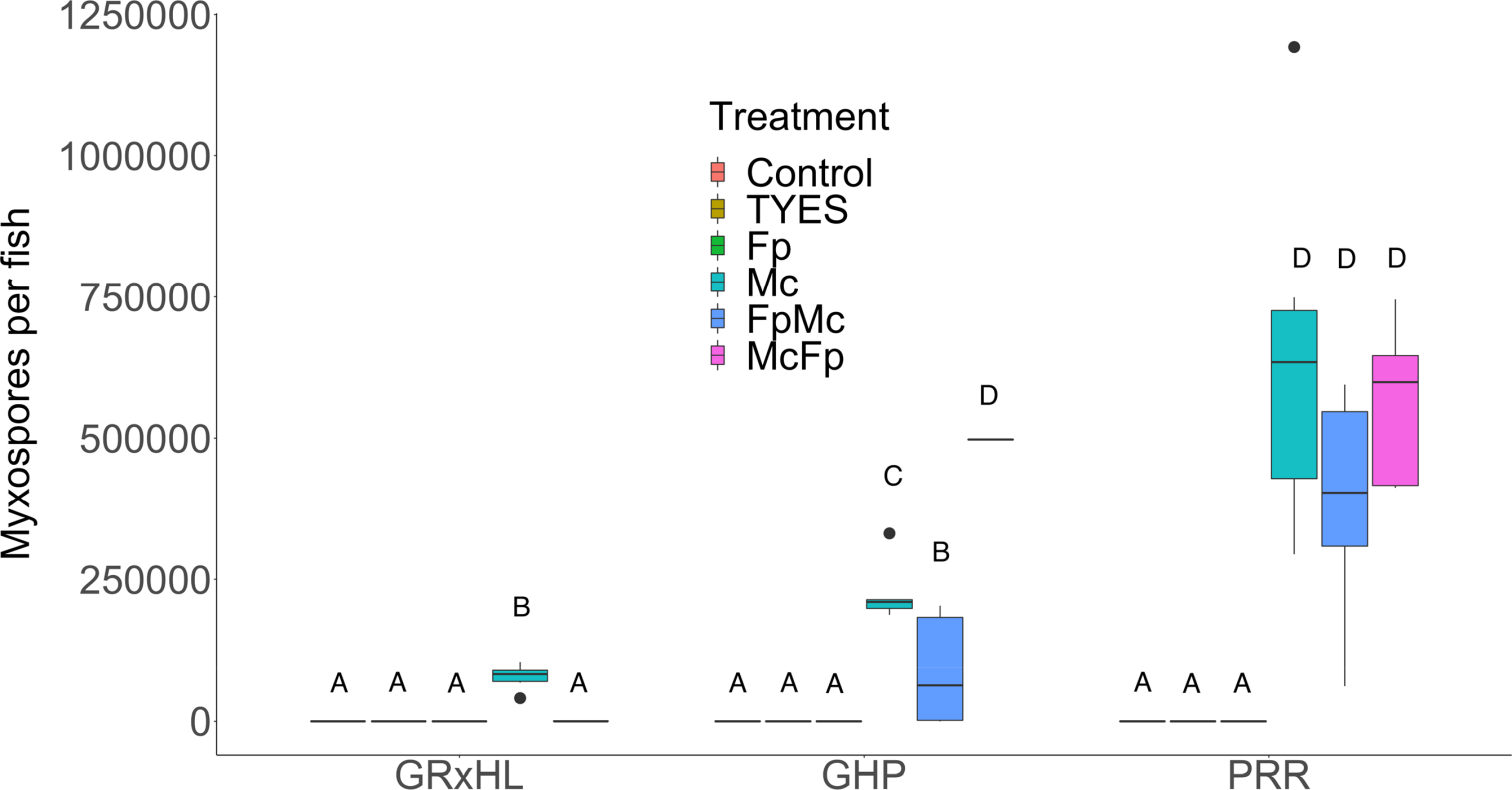
Mean myxospore count per fish by rainbow trout strain and treatment (TYES = mock injection, Fp = Flavobacterium *psychrophilum* only, Mc = Myxobolus *cerebralis* only, FpMc = exposed to *F. psychrophilum* followed by *M. cerebralis*, and McFp = exposed to *M. cerebralis* followed by *F. psychrophilum*) at the end of Experiment 1. No data available for GR × HL McFp treatment because of 100% mortality before the end of the experiment. Black lines within the boxes indicate the median of the distribution. Box and whisker plots with the same letter indicate no significant differences and box and whisker plots with different letters indicate statistically significant differences

As expected, none of the fish in the control, *F. psychrophilum*‐only, and TYES treatments had myxospores. Mean myxospore counts in the *M. cerebralis* exposure treatments (*M. cerebralis* only, *F. psychrophilum* followed by *M. cerebralis* exposure, and *M. cerebralis* followed by *F. psychrophilum* exposure) ranged between 556 (556; SE) and 645,201 (130,651; SE) per fish. The chi‐squared test indicated that there were differences between treatments exposed to *M. cerebralis* and those that were not exposed (χ^2^ = 106.45, *p*‐value <.01). The probability of having a myxospore count greater than zero was 99.9% when exposed to *M. cerebralis* compared to a probability of zero when not exposed to *M. cerebralis* (logistic regression). The negative‐binomial regression indicated a strain*treatment interaction (χ^2^ = 22.80, *p*‐value <.01). There were statistically significant differences among the PRR, GHP, and GR × HL when exposed to *M. cerebralis* only. The GR × HL strain developed the lowest number of mean myxospores (77,569 ± 9032; SE), followed by GHP (224,553 ± 21,704; SE) and then PRR (645,201 ± 130,651; SE; Figure 3). When fish survived exposure to *F. psychrophilum*, average myxospore counts were lower in the *F. psychrophilum* followed by *M. cerebralis* exposure treatment compared to the *M. cerebralis*‐only treatment (Figure 3). For the GHP, exposure to *M. cerebralis* followed by exposure to *F. psychrophilum* resulted in significantly higher myxospore counts.

#### 3.1.3 Clinical signs of whirling disease

Clinical signs of whirling disease (cranial deformities, spinal deformities, opercular deformities, lower jaw deformities, and blacktail) developed between two and three months post‐exposure. Both the GHP cross and PRR strain exhibited classic whirling swimming behaviour when fish were startled. The PRR strain had the most visible signs of whirling disease, with a significantly higher proportion of blacktail visible compared to the GR × HL and GHP and also showed extreme spinal deformities (Supplementary Materials; S1). Four out of the five clinical signs of disease (cranial, spinal, opercular, and lower jaw deformities) showed evidence of a strain by treatment interaction (*p*‐value <.01), with the PRR developing a higher percentage of these deformities in the *M. cerebralis* exposure treatments. None of the fish in *F. psychrophilum*‐only treatments developed a higher percentage of deformities than control fish.

### 3.2 Experiment 2

Mortalities started within the first two days post‐exposure for the ARS‐Fp‐R and the S‐Line strains compared to the other strains, which started between three and five days post‐exposure (Supplementary Materials; S2). There were no mortalities within the mock injection controls.

**FIGURE 4:**
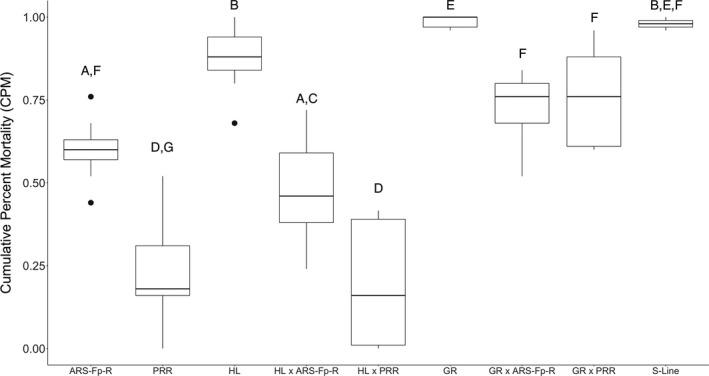
Cumulative per cent mortality by strain/cross for the fish exposed to *F. psychrophilum* only (mock injections not included) in Experiment 2. Black lines within the boxes indicate the median of the distribution. Box and whisker plots with the same letter indicate no significant differences and box and whisker plots with different letters indicate statistically significant differences

Chi‐squared tests indicated that fish weight did not have an effect on mortality among strains (strain*weight; χ^2^ = 9.68, *p*‐value = .08) and there was not an interaction between strain and exposure (χ^2^ = 0, *p*‐value = 1). However, there was a difference in mortality by both strain and exposure (χ^2^_strain_ = 544.95; χ^2^_exposure_ = 493.10; *p*‐value <.01). The TYES control groups showed no mortality associated with injection. The estimated probabilities of mortality for the strains in the *F. psychrophilum* exposure ranged from 19.3%–98.8%. The S‐Line, which was used as a positive control, showed expected high mortality (Figure 4). The S‐Line mortality was not different from that of the HL or GR strains (Figure 4), indicating that the *M. cerebralis*‐resistant strains were not resistant to *F. psychrophilum*, which was similar to experiment one. The PRR strain had significantly less mortality compared to the ARS‐Fp‐R strain (Figure 4), indicating that the PRR is more resistant to *F. psychrophilum* than ARS‐Fp‐R. The GR × ARS‐Fp‐R and GR × PRR showed less mortality than the GR strain and similar to that of the ARS‐Fp‐R (Figure 4) suggesting that some resistance was transferred from the *F. psychrophilum*‐resistant strains and indicating *F. psychrophilum* resistance in the F1 generation. The HL × ARS‐Fp‐R and HL × PRR showed less mortality than the HL strain (Figure 4) suggesting resistance was transferred from the *F. psychrophilum*‐resistant strains. In addition, the HL × ARS‐Fp‐R and HL × PRR showed the lowest mortality compared to the GR, HL, GR × ARS‐Fp‐R, and GR × PRR, suggesting that *F. psychrophilum* resistance can be passed on to F1 progeny. The HL × PRR and the PRR strains had the lowest mortality compared to the other nine, indicating the highest *F. psychrophilum* resistance among the strains.

## 4 DISCUSSION

The overall objective of our experiments was to evaluate the potential of developing rainbow trout that were resistant to both *M. cerebralis* and *F. psychrophilum*, suitable for use in both the hatchery system and for stocking in aquatic systems in which *M. cerebralis* is established. We investigated the consequences of infection with each pathogen and coinfection with both pathogens on two rainbow trout strains and one cross, and *F. psychrophilum* exposure effects in pure strains and F1‐generation crosses. It appears that some crosses might be useful in the development of rainbow trout that are resistant to both pathogens. However, others do not appear to have that potential. Strains known for their resistance to *M. cerebralis* were not resistant to *F. psychrophilum*, and vice versa, strains known for their resistance to *F. psychrophilum* were not resistant to *M. cerebralis*. The intermediate cross did not appear to be resistant to either pathogen. Despite the resistance characteristics of any given trout strain, coinfection led to an increase in average mortality for all strains compared to single‐pathogen exposure.

It appears that some rainbow trout crosses have greater promise for creating dual resistance than others. The results of the second experiment indicate that *F. psychrophilum*‐resistance can be maintained in first‐generation crosses, with the HL × PRR exhibiting the lowest mortality from *F. psychrophilum* exposure. These crosses may provide another management tool for fisheries managers, similar to the benefits of using *M. cerebralis*‐resistant rainbow trout. Use of the HL × PRR may reduce or eliminate the need for antibiotics, as the probability of mortality from *F. psychrophilum* exposure was less than 20%. Additionally, because the Harrison Lake rainbow trout originates from a wild rainbow trout population (Wagner et al., 2006), the HL × PRR may also show better survival and reproduction after being stocked compared to the PRR, because PRR are domesticated (Silverstein et al., 2009) and may not do well in the wild. We did not assess how the HL × PRR performed in the presence of *M. cerebralis* in the second experiment because additional evaluation was not possible due to time constraints involved with the development of the parasite. However, the HL × PRR may be a good candidate for the development of dual resistance to *F. psychrophilum* and *M. cerebralis*. Prior research has shown that the pure HL produce fewer myxospores than we observed in the GHP, GR × HL, and PRR (Schisler et al., 2011); additional research into *M. cerebralis* resistance in HL × PRR would be required to determine if the HL × PRR retains *M. cerebralis* resistance.

The GR × PRR or GR × ARS‐Fp‐R may still be a viable option for the development of dual resistance to both *M. cerebralis* and *F. psychrophilum*. The reduction in mortality associated with *F. psychrophilum* exposure was not as large as that seen in the HL × PRR but was still significant. Additionally, first‐generation rainbow trout cross progeny of the GR and the Colorado River rainbow trout (CRR) have shown high resistance to *M. cerebralis* (Fetherman et al., 2012) suggesting that both GR × PRR or GR × ARS‐Fp‐R may retain resistance to *M. cerebralis*. However, resistance to *M. cerebralis* in the GR × PRR and GR × ARS‐Fp‐R should still be confirmed.

Developing rainbow trout strains that are resistant to multiple pathogens will require an understanding of the immunological responses to each pathogen. Our research was not designed to address immunological responses; however, innate and acquired immune responses to each pathogen are undoubtedly complex (Cox, 2001). In dual infections such as ours, the effect of both infectious agents could be increased, suppressed, or one may be increased and the other suppressed, and the ultimate result may be hard to predict (Cox, 2001). Specific genes and innate resistance have been reported for rainbow trout immune response to *M. cerebralis* (Baerwald et al., 2008; Saleh et al., 2019). Exposure to *M. cerebralis* results in the activation of the cytokine gene IL‐1β that is a part of the innate immune system in fishes (Baerwald, 2013). The IL‐1β gene is also associated with immune responses to the bacterial pathogen *Yersinia ruckeri* and involved in resistance to *Aeromonas salmonicida* (Baerwald, 2013; Hong et al., 2003; Raida et al., 2011), indicating that achieving an innate response to both pathogens through selective breeding may be possible. In our study, we injected *F. psychrophilum* to ensure that all individuals received the same dose of bacteria and because immersion exposure to *F. psychrophilum* results in considerable variation in exposure depending on experimental circumstances (Avila, 2021; Garcia et al., 2000; Langevin et al., 2012). Injection bypasses important innate immune systems of fishes, particularly those in the skin and mucus (Makesh et al., 2015; Nematollahi et al., 2003), and our results may be influenced by our experimental protocol. Although we choose the injection protocol, many of the genes identified in fish immune systems also have a role in acquired fish immune responses (Baerwald, 2013), and it was expected that the immune system would still be activated in response to exposure to *F. psychrophilum* despite the exposure method. Clearly, future development of dual resistance will require studies on the immune responses to each pathogen and the activation of these immune responses regarding the order in which exposure to each pathogen occurs.

Although it appears that dual resistance may be possible with some strains, the lack of response in others indicates that dual resistance may be difficult to develop. The GHP showed no resistance to either pathogen as single exposures to *F. psychrophilum* and dual exposures to *F. psychrophilum* and *M. cerebralis* resulted in both high mortalities (>75%) and high myxospore counts. High mortality and high myxospore counts indicate that the GHP is not a good candidate for developing dual resistance, particularly because it seems to have lost resistance to both pathogens. Currently, it is unknown which genes provide resistance to *F. psychrophilum* (G. Weins, personal communication, February 16, 2021). Development of the *F. psychrophilum*‐resistant rainbow trout used selective breeding at the USDA‐ARS NCCWA (Hadidi et al., 2008; Leeds et al., 2010; Silverstein et al., 2009; Wiens et al., 2013). However, genetic parentage analyses were not done, and the mechanism of genetic resistance may depend on the specific parent strains and genes that allow for disease resistance. The GR is highly resistant to *M. cerebralis* with 9 ± 5 genes estimated to confer genetic resistance (Fetherman et al., 2012). These genes are additive in their effect (Fetherman et al., 2012) and therefore if all genes are not passed onto future generations this may result in lower resistance to *M. cerebralis* in outcrosses with *F. psychrophilum*‐resistant fish. A possible reason that the GHP showed little to no resistance to *F. psychrophilum* is that the GR genes that confer resistance to *M. cerebralis* may negatively interact with the genes that infer *F. psychrophilum* resistance (Fraslin et al., 2020; Lhorente et al., 2014).

We included the ARS‐Fp‐R strain to determine if it had higher *F. psychrophilum* resistance than the PRR, because it had undergone more generations of selection (three for the PRR versus five for the ARS‐Fp‐R) and was predicted to show similar or lower mortality due to the generational differences in *F. psychrophilum*‐resistance selection (G. Weins, personal communication, February 16, 2021). The lower mortality in the PRR indicates that it has higher resistance to *F. psychrophilum* and additional selection in the ARS‐Fp‐R rainbow trout strain did not confer greater resistance. One explanation is that there were other environmental variables not accounted for, for example, transportation of eyed eggs from West Virginia to Colorado may have induced additional stress due to temperature and pressure changes, resulting in increased mortality. Both the ARS‐Fp‐R and the S‐Line, which were spawned in West Virginia and then sent to Colorado, showed mortality beginning around day two of the experiment which is slightly earlier than the traditional time frame seen in all other rainbow trout strains. Another explanation for the differences in mortality between the strains is the continuous exposure to *F. psychrophilum* in the CPW hatchery system that may have allowed the PRR to develop increased resistance compared to the ARS‐Fp‐R. Similar continuous exposure is believed to have produced the *M. cerebralis* genetic resistance in the GR strain (Hedrick et al., 2003). Based on these results, there is no need to replace the current PRR brood stock with another *F. psychrophilum*‐resistant brood stock that experienced more generations of selection in the CPW hatchery system.

A concerning and unexpected observation was the relatively high average myxospore counts for the *M. cerebralis*‐resistant GR × HL. The high number of myxospores found in the GR × HL indicates a loss of resistance and could be attributed to backcrossing or lack of exposure to the parasite. Outcrossing and/or backcrossing may have occurred in the hatchery and resulted in decreased genetic resistance to *M. cerebralis*. The observed myxospore counts were roughly the same as those seen in second‐generation backcrosses of F1‐generation GR × CRR with the CRR (Fetherman et al., 2012). A loss of resistance could also be the result of the absence of *M. cerebralis* in the hatchery system. In a single hatchery generation, the expression of hundreds of genes in rainbow trout can be altered, resulting in selection of traits that are beneficial in the hatchery but not in the wild (Christie et al., 2012, 2016). The absence of the parasite could therefore reduce selection for resistance to *M. cerebralis* given that those genes are not needed for survival in the hatchery environment. The loss of resistance to *M. cerebralis* in the GR × HL strain is concerning for future management and reintroduction efforts. Stocking rainbow trout that are susceptible to the parasite could result in less successful survival and recruitment (Avila et al., 2018). Additionally, these fish could produce high numbers of myxospores, which may lead to increased *M. cerebralis* in wild systems.

The PRR strain showed no resistance to *M. cerebralis* and had 3.45 times more myxospores than the highly susceptible CRR (Fetherman et al., 2011). The GHP had similar numbers of myxospores as the CRR, also indicating no genetic resistance to *M. cerebralis*. High numbers of myxospores and high mortality indicated that neither the PRR nor GHP strains are good candidates for stocking into *M. cerebralis‐*positive waters. Stocking these strains could result in increased *M. cerebralis* and loss of fish due to *M. cerebralis* infection.

Co‐infection with *F. psychrophilum* and *M. cerebralis* increased CPM for every rainbow trout strain. Similar increases in mortality have been seen with parasite and bacterial co‐infections compared to single‐pathogen exposure of rainbow trout in previous experiments (Bandilla et al., 2006; Busch et al., 2003; Schisler et al., 2000). Ma et al. (2019) also showed higher mortality in rainbow trout with co‐infections of *F. psychrophilum* and infectious hematopoietic necrosis virus (IHNV), compared to those infected with a single pathogen. Currently, it is not known what factor(s) increase mortality due to co‐infection or the specific interactions between *M. cerebralis* and *F. psychrophilum*. Co‐infections are common within the hatchery and wild environments due to exposure to heterogeneous infectious pathogens (Kotob et al., 2016; Ma et al., 2019), and reduced post‐stocking survival may result from co‐infection in hatchery or wild environments. Reducing disease exposure in hatcheries by changing or improving husbandry protocols may not only reduce disease outbreaks but increase long‐term survival within the hatchery and/or post‐stocking survival.

Our research demonstrates that the development of dual resistance to both *F. psychrophilum* and *M. cerebralis* is attainable but is dependent on the specific rainbow trout strains that are used, and presumably the genetic compatibility of their individual resistance. The GHP cross was not resistant to either pathogen; however, increased resistance to *F. psychrophilum* in the HL × PRR and HL × ARS‐Fp‐R suggests that dual resistance may be possible. Further research will be needed to evaluate *M. cerebralis* resistance of the HL × PRR or HL × ARS‐Fp‐R crosses. Dual resistance will benefit both aquaculture production and management of wild fisheries and has implications for the management and protection of other salmonid fishes.

## CONFLICT OF INTEREST

The authors have no conflict of interest to declare.

## Supporting information

Fig S1‐S2

## Data Availability

The data that support the findings of this study are available from the corresponding author upon reasonable request.
