# Supplementary material for: Dual resistance to Flavobacterium psychrophilum and Myxobolus cerebralis in rainbow trout (Oncorhynchus mykiss, Walbaum)
Source: J Fish Dis. 2022 Mar 8;45(6):801–13. doi: 10.1111/jfd.13605 (PMC9314901; doi:10.1111/jfd.13605)
Supplement: Supplementary file 1 — Fig S1‐S2 [file JFD-45-801-s001.docx]

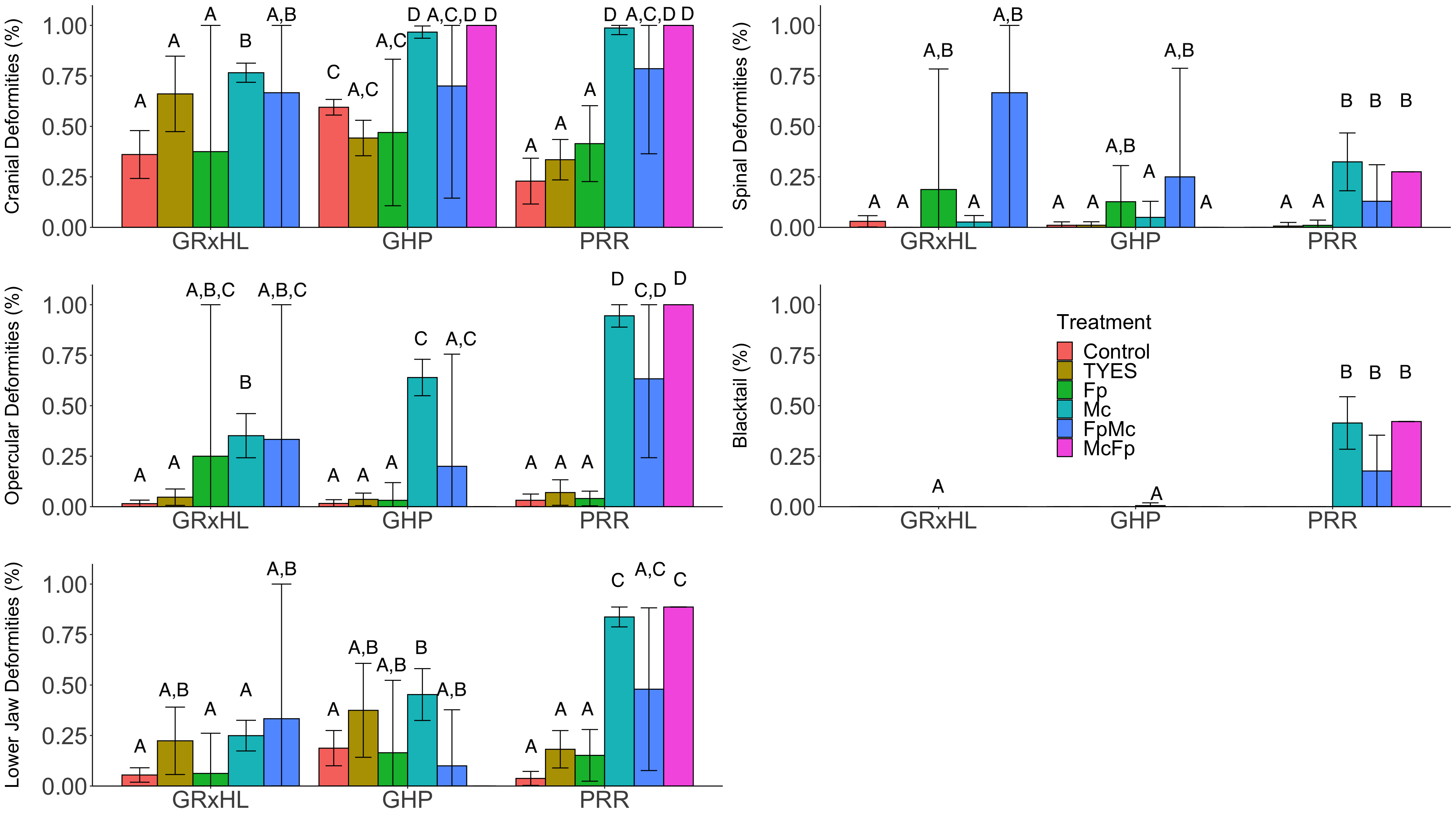


S1. Percent of deformed individuals (standard error bars) exhibiting cranial deformities, spinal deformities, opercular deformities, lower jaw deformities, and blacktail by strain and treatment (TYES = mock injection, Fp = *Flavobacterium psychrophilum* only, Mc = *Myxobolus cerebralis* only, FpMc = exposed to *F. psychrophilum* followed by *M. cerebralis*, and McFp = exposed to *M. cerebralis* followed by *F. psychrophilum*) at the end of Experiment 1. No data available for the GRxHL McFp treatment because of 100% mortality before end of experiment.


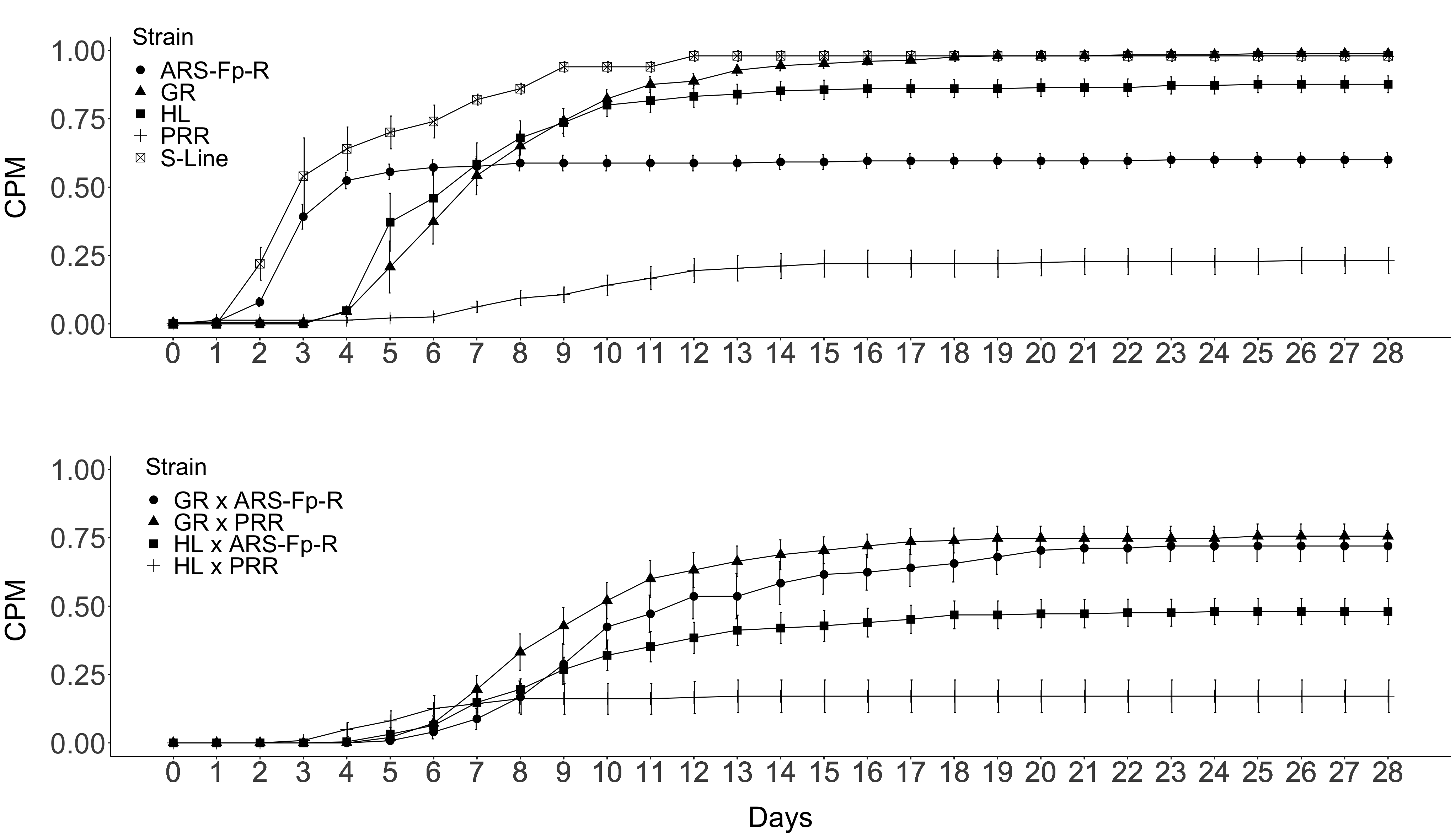


S2. Cumulative percent mortality (CPM; standard error bars) in the first 28 days of Experiment 2 for each strain or cross exposed to *Flavobacterium psychrophilum*.
